# A pilot short-term study of feasibility, acceptability and preliminary efficacy of 3-stage 8-session 4-week group therapy-based narrative intervention in 17 improved hospitalized female schizophrenia patients in southern China

**DOI:** 10.3389/fpubh.2025.1594471

**Published:** 2025-06-10

**Authors:** Qian Zhou, Yuhua Wei, Zaifei Huang, Yue Zhao, Sujiao Qin, Xuewei Wei, Lianou Wei, Da Huang, Mingkang Qin, Lingjun Zeng, Fengqiong Qin, Yuting Huo

**Affiliations:** ^1^Guangxi Zhuang Autonomous Region Brain Hospital, Liuzhou, China; ^2^Liuzhou Liujiang District Hospital of Traditional Chinese Medicine, Liuzhou, China

**Keywords:** self-esteem, psychological capital, schizophrenia, narrative nursing, self-stigma

## Abstract

Self-stigma has been consistently cited as a major obstacle to recovery-related outcomes among patients with schizophrenia. To examine the feasibility, acceptability, and preliminary efficacy of the group-based narrative intervention for improving self-stigma, self-esteem and psychological capital in hospitalized patients with schizophrenia, a case-series study was conducted from March to May 2023 in a closed psychiatric ward of a specialized hospital in mainland China. Feasibility was assessed by examining rates of recruitment, retention, and protocol adherence. Acceptability was assessed through the therapist’s and patients’ feedback about the intervention. Changes in the levels of self-stigma, self-esteem, and psychological capital perceived by patients were measured before and after 4 weeks of intervention. Rates of enrolment (85%) and completion of intervention sessions and study procedures (100%) were excellent, demonstrating high rates of feasibility among these patients in the local setting. The feedback from participants and the therapist about satisfaction, helpfulness, and difficulty of the intervention was largely positive, demonstrating high rates of acceptability. And the results indicated significant improvements in patients’ self-reported self-stigma, self-esteem, and psychological capital (change in *T* = 3.872, *p* = 0.001; *T* = −6.308, *p* < 0.001; *T* = −2.895, *p* = 0.011, respectively). The study provided a structured intervention program for clinical care to reduce self-stigma and promote positive recovery outcomes for inpatients with schizophrenia.

## Introduction

Schizophrenia, characterized by profound disruptions in an individual’s thinking, perception, speech, and behavior causes psychosis and is associated with considerable disability (1). Schizophrenia significantly contributes to the global burden of disease, affecting approximately 1% of the global population, and is one of the top 10 causes of disability worldwide (2). Stigma, defined as the devaluation of a group or individual on the basis of a characteristic that is discredited by society, is highly prevalent among people with schizophrenia and has been consistently cited as a major obstacle to recovery and quality of life among those people (3, 4). A systematic review with over 25,000 participants reported that 35% of persons diagnosed with schizophrenia-spectrum disorders overall demonstrated elevated self-stigma (5).

Public stigma refers to negative stereotypes, prejudices, and discrimination against individuals by the outside world, whereas self-stigma occurs when individuals agree with and internalize these negative stereotypes and prejudices about their devalued conditions and suffer numerous negative consequences as a result (6–8). With respect to illness and pathologies, biomedical discourse, and public stigma, people with schizophrenia often perceive themselves as “dangerous,” “incompetent,” as well as “worthless” and believe that they have become a burden on their family and society (9, 10). This may lead to the idea that their inner self is the cause of the problem, which could result in negative self-identity (11). The public stigma embedded in the Chinese-dominant culture further causes negative self-identity among people with schizophrenia (11, 12), resulting in the internalization of negative stereotypes, self-stigma and its adverse effects (6, 13).

Stigma has been implicated in worsening outcomes for people with schizophrenia (13, 14). Patients who experience self-stigma are unable to maintain positive self-identity, resulting in reduced self-esteem, self-efficacy, and self-worth (15, 16). Hence, patients tend to adopt negative coping strategies such as avoidance and withdrawal, which are associated with reduced treatment compliance and diminished social functioning and quality of life, and even make patients feel hopeless and increase the risk of suicide (13, 17, 18). Despite the substantial evidence for the negative effects of self-stigma, the development of anti-stigma interventions is a relatively limited area of research (3, 11, 19). As a result, there has been a shift in interest toward developing interventions to address and ameliorate self-stigma.

The evidence on the effectiveness of narrative therapy among patients with serious mental illness is promising but limited (10, 20–22). The primary focus of narrative therapy is people’s expression of their experiences, which focuses on meaning-making and transforming one’s life story from a more positive and appreciative perspective (23), instead of focusing on the disabilities and limitations from the traditional problem-focused model (24). It holds that problems separate from the individual could be solved through therapeutic conversations, and assumes that everyone has inner resources, skills, and competencies to accommodate transitions and challenges in their lives (24). During therapeutic conversations, therapists help participants to deconstruct their problem-saturated story from the illness experience, coconstruct their inner strengths and beliefs from previous life challenges, and reconstruct their positive identity (24). In group practice, the narrative process allows participants to gain supportive feedback and experiences, skills, and knowledge from peers and provides ongoing outsider witnesses to strengthen individual positive self-identity (18, 25, 26). Despite the limited evidence, it has been demonstrated that group-based narrative interventions could improve self-esteem in patients with severe mental illness and reduce their self-stigma (9, 10, 19, 25). In addition, the targeted interventions were also supposed to focus on improving psychological capital (e.g., self-esteem, self-efficacy, resilience, hope and optimism), which were found to positively influence stigma resistance and counteract internalized stigma (11, 12, 27).

Given the negative consequences of self-stigma in the recovery process among patients with schizophrenia, there seems to be a need to expand the current evidence concerning narrative intervention. To date, there has been no attempt to apply a group-based narrative intervention among hospitalized patients with schizophrenia in the Chinese mainland (20, 28). Therefore, the aim of the current study was to pilot examining the feasibility and acceptability of a group-based narrative intervention consisting of eight sessions for hospitalized patients with schizophrenia. We also evaluated the preliminary efficacy in reducing self-stigma and improving the self-esteem and psychological capital of patients.

## Methods

### Design

A case series study was employed, as the first stage of piloting within the process of intervention development, informed by the Medical Research Council recommendation framework for the development of complex interventions (29). Ethical approval was obtained from the Committee of Ethics at the Hospital before the study procedures began (No. 2022-021). Written informed consent was obtained from all patients. All methods were performed in accordance with the 2013 Declaration of Helsinki.

### Setting

The study was performed in the closed psychiatric ward which only admits female patients with mental disorders in the local specialized hospital. It is the largest specialized hospital for mental and psychological disorders in Liuzhou, a city in southern China. The study hospital, with medical treatment, teaching, scientific research, prevention, rehabilitation, and medical identification, offers advanced health services for people with mental and psychological disorders from regional and surrounding areas (30). The psychiatry department consists of 15 psychiatric wards, which service an average of 700 hospitalized patients per day, seven of which are under closed management. The ward in which the study was conducted has a capacity of approximately 70 beds per ward, servicing more than 60 hospitalized patients per day.

### Participants

A convenience sample of hospitalized patients in the closed psychiatric ward from March to May 2023, where a total of 17 eligible patients were employed, was used. The inclusion criteria for selecting the subjects were as follows: (1) diagnosed with schizophrenia who scored 60 points and below on the Positive and Negative Syndrome Scale (PANSS); (2) aged 18–60 years; (3) had sufficient cognitive and communicative ability to participate; and (4) provided informed consent and volunteered to participate in the study. Patients were excluded if they (1) were co-diagnosed with other mental disorders; (2) had a serious physical illness; (3) had severe cognitive impairment or symptoms such as excited impulsivity, suicidal ideation with intent or plan, or reported recent self-harm; or (4) had poor compliance or withdrawal.

### Procedures

The research team was established to implement the procedures successfully.

The principal researcher (QZ), experienced in individual narrative and group counseling and two advanced clinical consultant psychologists (YW and ZH) were involved in the development of the intervention. The principal researcher carried out the program training of the intervention and techniques to the other researchers and assessed them for quality. The narrative therapist (XW) certified with psychological consultation delivered the intervention, which was supervised by the advanced clinical consultant psychologists (ZH and YZ). The other two researchers (LW and DH) who did not work in the ward were assigned to complete the screening and organize the distribution and collection of questionnaires, which aimed to maintain the rights of the participants to not consent to the study.

Potential participants were first screened via the electronic medical records of the hospitalized patients. If eligible on the basis of this screening, they were invited for interview screening. Following the interview screening, eligible participants were invited for the research project. The written questionnaires, together with a cover letter of informed consent that assured the confidentiality of information and autonomy of the participants who anonymously participated, were distributed to the eligible participants. Additionally, a five-minute long WeChat video, which illustrated the background, purpose and significance of the study, which aimed to arouse the interest and resonance of patients, was delivered to each eligible participant. Moreover, it explained the meanings of the questionnaires with instructions on how they should be completed and how to manage the information and data of the participants.

### Intervention

**Figure 1:**
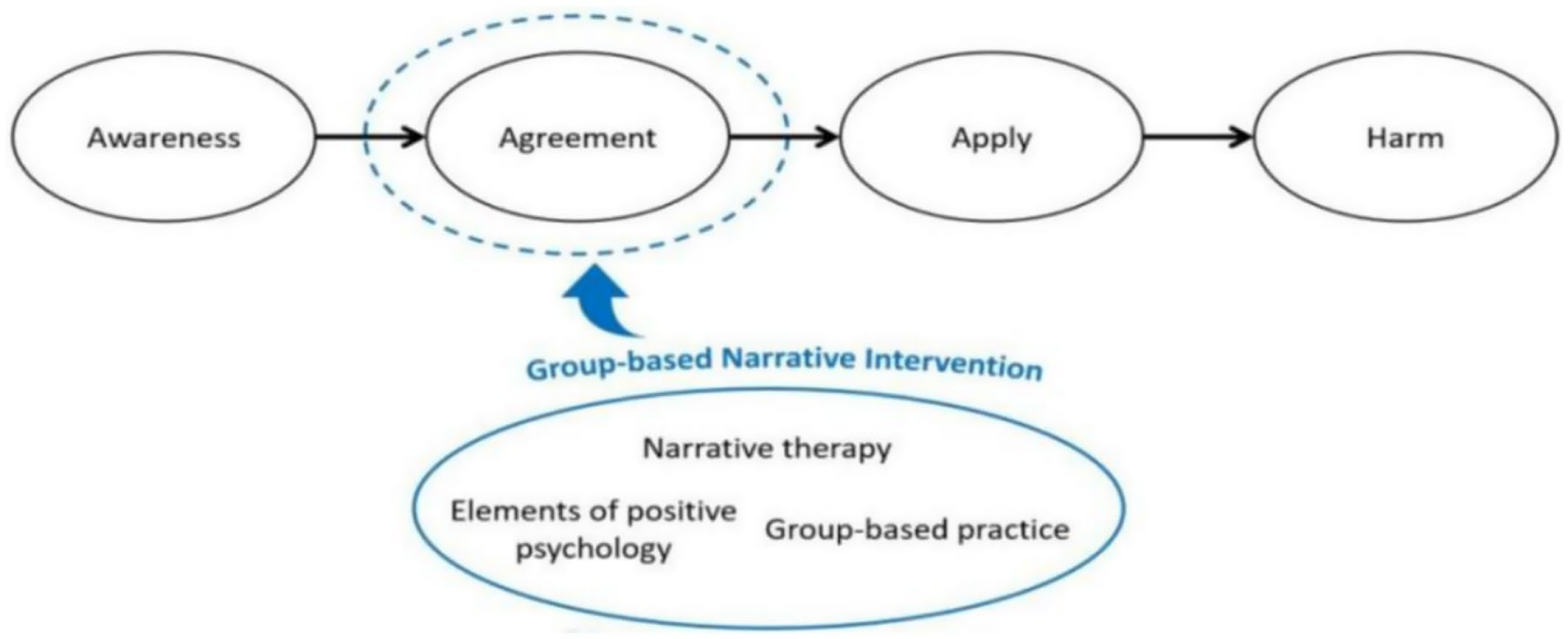
Theoretical framework of the intervention on the basis of the stage model of self-stigma.

The theoretical framework of the narrative intervention was developed by the stage model of self-stigma and relevant evidence (6, 11, 12, 27, 31–34), as shown in Figure 1. According to Corrigan’s model (6), the formation and influence process of self-stigma includes four stages: awareness, agreement, application and harm. When people perceive and identify with the public negative stereotype about their disease, the negative stereotype is then internalized to be self-prejudiced and self-discriminated, ultimately resulting in negative emotional reactions and behavioral responses. Therefore, the agreement of negative stereotypes plays a key role in the formation of self-stigma, which the current intervention focuses on. Narrative intervention contributes to the reconstruction of their positive identity. Additionally, it was based on group psychotherapy, where the other participants were regarded as outsider witnesses used to strengthen their preferred identity. Moreover, the intervention also targeted to improve the elements of positive psychology, such as self-esteem and positive psychological capital, which have been shown to be protective factors for reducing patients’ self-stigma and enhancing resistance to stigma.

**Figure 2:**
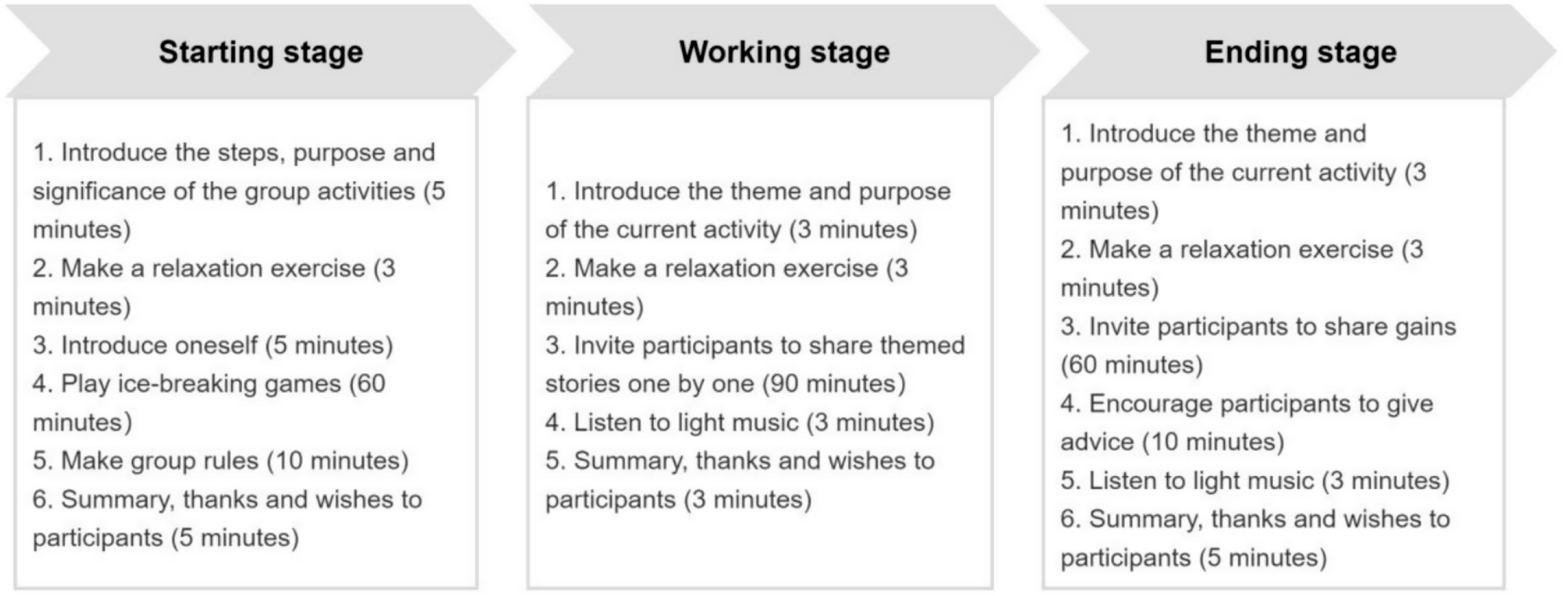
Operation procedure of the group-based narrative intervention.

The group-based narrative intervention was composed of three stages, for a total of eight sessions, and the components of the intervention are shown in Table 1. The operation procedure of each stage is shown in Figure 2. The sessions were implemented by the narrative therapist in a separate psychotherapy room, where the outside door had a sign that read “Be quiet, meeting in process,” approximately 90 min each session, twice a week, and lasted for 4 weeks. During each session of the working stage, participants were invited to share thematic stories guided by the therapist, with the principle of being voluntary, nonjudgmental, respectful, and confidential.

**Table 1:** Components of the group-based narrative intervention.

Stages	Themes of sessions	Components	Objectives
Starting stage	We are together	Play ice-breaking games Make group rules together with participants	Build up supportive relationships, form interactive and companionable atmosphere
Working stage	That disease and me	Invite participants to name or describe the disease Invite participants to talk about the impact of that disease on them, and evaluate the impact	Externalize the disease, separate a person’s identity from the disease, reduce negative self-identity
	Behind that disease	Invite participants to share the story about how that disease develops	Deconstruct the problem-saturated stories, help understand the disease attribution
	The different me	Invite participants one by one to share one story they are most proud of and its impact, and guide the participant to make a self-evaluation Invite the other participants to share their feelings, including what touched them most in the last story, what imagery and resonance it gave, and how it changed them	Reconstruct positive self-identity by exploring alternative stories, and help improve self-esteem and self-efficacy
	Witness the most beautiful self	Invite participants one by one to share how they got through the toughest encounters and its impact, and guided the participant make a self-evaluation again Invite the other participants to share their feelings, including what touched them most in the last story, what imagery and resonance it gave, and how it changed them	Reconstruct positive self-identity by exploring alternative stories, and help perceive individual resilience
	Praise my companions	Invite participants to share the story that touched her most in the other participants’ sharing and its impact on her, and express her praise and gratitude	Strengthen positive self-identity by external witness
	A trans-dimensional dialog	Invite participants to share their dreams Invite participants to hypothesize that the dreams come true, what they would like to say to themselves in the current predicament	Help build hope and optimism, and help to turn into positive behaviors
Ending stage	Departure is a new voyage	Invite participants to share their gains Encouraged participants to give advice	Summarize experiences

### Feasibility and acceptability objectives

Feasibility was assessed by examining rates of recruitment, retention, and protocol adherence. Acceptability was assessed through participants’ feedback.

### Fidelity

Each session was audio-recorded and listened to by the therapist’s supervisors to assess fidelity, which guarantees that the intervention could be delivered adequately by the therapist. Session recordings were rated a dichotomous yes/no score for whether the therapist adhered to the session protocol. Protocol violations were recorded, and were fed back to the therapist.

### Measures

Demographic data questionnaire and the standardized scales were completed at pre-intervention. The levels of self-stigma, self-esteem, and psychological capital perceived by patients were reevaluated after 4 weeks of intervention.

Demographic data questionnaire. Demographic information included age, marital status, educational background, employment status, and disease duration.

Self-Stigma Scale-Short Form (SSS-S). The scale consists of 9 items with a 4-point Likert scale (1 = strongly disagree to 4 = strongly agree). It contains three subscales: self-stigmatizing cognitions, self-stigmatizing affect, and self-stigmatizing behaviors. The higher the score is, the greater the level of self-stigma perceived. The Cronbach’s alpha coefficients for the total scale and dimensions ranged from 0.80 to 0.91 (35).

Self-Esteem Scale (SES). The scale was used to assess overall feelings about self-worth and self-acceptance. It contains 10 items with a 4-point Likert scale (1 = strongly disagree to 4 = strongly agree), of which five items provide a negative statement and are reverse scored. The total scores range from 10 to 40. Higher scores indicate greater levels of self-esteem perceived by patients. The Cronbach’s alpha coefficient of the total scale was 0.88 (36).

Psychological Capital Questionnaire (PPQ). The questionnaire contains 26 items divided into four domains: self-efficacy, resilience, optimism, and hope. Each item is scored from 1 (strongly disagree) to 7 (strongly agree), with higher scores indicating a higher level of psychological capital. The total scores range from 26 to 182. The Cronbach’s *α* coefficients for the total scale and dimensions ranged from 0.76 to 0.90 (37).

### Feedback

Qualitative feedback was collected from the participants, therapist, and supervisors after intervention delivery was completed, with open-ended questions about the content, process, and usefulness of the intervention. During the interviews, the principal researcher maintained a neutral attitude and did not express personal judgments, beliefs, or understanding.

### Statistical analysis

Data analysis was performed via the Statistical Program for Social Sciences version 22.0. The Kolmogorov–Smirnov test was used to assess the normality of the data. The count data are presented as frequencies and composition ratios. The measurement data conforming to normal distribution or approximate normal distribution were reported as means and standard deviations. A paired-samples *t* test was performed to compare the measured data before and after the intervention. Statistical significance was specified at 95% confidence intervals and two-tailed *p* values of less than 0.05 for all tests.

## Results

### Participant characteristics

A total of 17 patients were enrolled in the study and completed the intervention. Table 2 shows the demographic and clinical characteristics of all the participants.

**Table 2:** Demographic characteristics of the participants.

Characteristics		Participants, No. (%) or Means ± Standard Deviations
Age	18–35 years old	10 (58.82%)
	≥36 years old	7 (41.18%)
Marital status	Married or cohabiting	6 (35.29%)
	Unmarried or living alone	7 (41.18%)
	Divorced/widowed	4 (23.53%)
Educational background	Junior high school and below	8 (47.06%)
	High school and above	9 (52.94%)
Employment status	Employed	6 (35.29%)
	Unemployed	11 (64.71%)
Disease duration	5 years and below	6 (35.29%)
	6–9 years	4 (23.53%)
	10 years and above	7 (41.18%)
Scores of positive and negative syndrome scale	Total scores	48.18 ± 6.356
	Positive symptom scores	11.06 ± 2.221
	Negative symptom scores	13.18 ± 2.186
	General psychiatric symptom scores	23.94 ± 2.512

### Feasibility

The recruitment target was reached in a two-week period with two researchers working part-time. The researchers attempted to reach 20 patients for the study, while 17 of those (85%) assessed were deemed eligible and enrolled in the study. The retention rate was high, with no sample lost during the intervention, and all participants completed the intervention and measures. All participants completed the questionnaires fully before and after the intervention.

### Acceptability

In the feedback interviews, all the participants expressed that the intervention was helpful and satisfactory. Most participants (15 patients [88.24%]) found 8 sessions to be the right length and found the intervention to contain the right amount of information. In addition, 14 patients (82.35%) indicated that the content of the intervention was easy to understand, whereas 3 participants (17.65%) who reported some degree of difficulty did not specify the aspects that were difficult to understand. Moreover, approximately half of the participants reported that they would like to participate in more sessions, such as this, during their hospitalization. Additionally, the feedback from the therapist about the experience of delivering the intervention was positive, particularly for the modular structure, enabling sessions to be delivered flexibly.

### Fidelity

For the fidelity of the intervention, 100% consistency in the pacing, introduction to purpose of intervention and the delivery of intervention components were observed, meeting the benchmarks for treatment fidelity.

### Changes in measures

A total of 17 patients were enrolled and completed final measures and feedback. There were significant improvements in self-stigma, self-esteem, and psychological capital from pre- to post-intervention (change in *T* = 3.872, *p* = 0.001; *T* = −6.308, *p* < 0.001; *T* = −2.895, *p* = 0.011, respectively). Reliable improvements in self-stigma were made by 13 participants (76.47%) at post-intervention. Reliable recovery in self-esteem was found for 15 participants (88.24%) at post-intervention. With respect to psychological capital, 12 participants (70.59%) made reliable improvements at post-intervention. The results of the statistical analysis of the pre- and post-operative changes in the outcomes via standardized questionnaire scores are presented in Table 3.

**Table 3:** The self-stigma, self-esteem and psychological capital scores of patients before and after the intervention (means±standard deviations).

Evaluation indicators	Before the intervention	After four-week intervention	T*	*P*
Self-stigma scores	23.00 ± 6.114	16.41 ± 4.388	3.872	0.001
Self-esteem scores	26.29 ± 3.584	31.35 ± 4.271	−6.308	0.000
Psychological capital scores	110.94 ± 24.283	133.06 ± 15.159	−2.895	0.011

*T is a statistic value analyzed by paired-samples *T* test.

## Discussion

In the context of this case series study, we examined the feasibility, acceptability, fidelity, and preliminary efficacy of the group-based narrative intervention composed of eight sessions, which was the first attempt to develop and apply a group-based narrative intervention for hospitalized patients with schizophrenia in mainland China. Randomization was not included because the purpose of the current study was to undertake primary data collection related to the feasibility and acceptability of the novel intervention. The results suggested that the group-based narrative intervention and its procedures were feasible, acceptable, had high rates of intervention fidelity, and demonstrated potential efficacy at improving targeted outcomes. The rates of enrolment (85%) and completion of intervention sessions and study procedures (100%) were outstanding, demonstrating high rates of feasibility among these patients in the local setting. The feedback from the participants and the therapist about the level of satisfaction, helpfulness, and difficulty of the intervention was largely positive, demonstrating high rates of acceptability. Additionally, some participants hoped that there were more activities like it to participate in.

Most promising, the study results indicated significant improvements in participants’ self-reported self-stigma, self-esteem, and psychological capital, which suggested that the group-based narrative intervention might be an effective intervention at improving self-stigma and the related outcomes for hospitalized patients with schizophrenia. These findings were consistent with those of previous studies supporting the effectiveness of group-based narrative intervention (9, 10, 19, 25). It appeared that the narrative intervention process affirms and reconstructs a self-identity of participants that was possibly troubled by its problem, improving their self-perceived cognitive about the disease and rejecting a negative self-identity due to public stigma (38). Therefore, the internalization of perceived stigma was reduced. Simultaneously, on the basis of group practices, positive self-identity was further strengthened by ongoing external witnesses with other participants. Additionally, its effectiveness was enhanced by intervening with elements of positive psychology, increasing the resistance of public stigma (11, 27, 39, 40).

For the process of the intervention, sessions 2 and 3 were performed to help participants understand the attribution of the disease and reduce negative self-attribution about the disease and the problem-saturated identity. This study provides a new perspective for participants in understanding disease and its effects instead of providing a view of their disabilities and limitations. By exploring alternative stories during sessions 4–6, it focused on developing self-esteem, self-efficacy, and resilience and reconstructing the positive self-identity of participants as opposed to the negative self-identity. In addition, session 7 further focused on increasing the hope level of participants to regain the power to move closer to their hopes and dreams, which helped to turn their positive self-identity into positive behaviors to cope with present difficult situations. By exploring narratives from participants’ past experiences, the patients in the study had the opportunity to appreciate their strengths, resources, and capabilities and develop problem-solving skills to overcome challenges. Hence, the participants’ perceptions of their efficiency and worthiness were greatly increased. This narrative intervention poses an alternative methodology to counteract negative stereotypes, providing participants with restructuring cognitive and adaptive coping tools.

## Limitations

Despite promising results, there are limitations that must be acknowledged when interpreting the results of this study. First, this finding is only reflective of the practice among hospitalized female patients with schizophrenia in the local setting from southern China. As a result, it is unclear whether the intervention is applicable to male patients and other regions. Second, the sample size and pre-post design limit the ability to examine the efficacy of the group-based narrative intervention. It is not possible to conclude that the observed changes in the measures were associated with the intervention. Finally, the feasibility of therapists new to the intervention being able to deliver it adequately in real-world settings, including less intense supervision, requires investigation.

## Future research

In future research, the next stage of investigation should include randomization and blinded researchers to provide a more reliable estimate of the effect size associated with the intervention. Further investigations should examine the feasibility, acceptability, and potential effectiveness of the intervention in the multiple-spot context with multiple samples. A longer follow-up investigation post-intervention would enable exploration of the longer-term effects of the intervention.

## Conclusion

Preliminary evidence from this case series study revealed that the group-based narrative intervention was feasible and acceptable in mainland China. Furthermore, significant improvements were observed in reducing self-stigma and improving self-esteem and psychological capital among these patients. However, these findings indicated the need for further investigations in future research. Even so, a pilot study of group-based narrative practices proposed a viable perspective to improve self-stigma and its potentially negative consequences for people with schizophrenia.

## Relevance for clinical practice

This was the clinical practice to carry out a pilot study of a group-based narrative intervention tailored for hospitalized patients with schizophrenia in mainland China, aiming to improve their perceived self-stigma, self-esteem, and psychological capital. On the one hand, the study method provided a perspective for the development of complex interventions based on the Medical Research Council recommendation framework in the clinical care of patients with mental illness. On the other hand, this study provided a structured intervention program for clinical care to reduce self-stigma and promote positive recovery outcomes for inpatients with mental illness.

## Data Availability

The data analyzed in this study is subject to the following licenses/restrictions: aggregated data is provided within the manuscript, while individual data is unavailable due to ethical restrictions. Requests to access these datasets should be directed to Yuting Huo, 757514913@qq.com.
